# Immunogenicity of a CXCL8-based biopharmaceutical drug candidate in comparison to its wildtype form

**DOI:** 10.3389/fimmu.2025.1679409

**Published:** 2025-12-16

**Authors:** Elisa Talker, Tanja Gerlza, Philippe Stas, Christina Trojacher, Tiziana Adage, Andreas J. Kungl

**Affiliations:** 1 Institute of Pharmaceutical Sciences, University of Graz, Graz, Austria; 2 AlgoNomics, Applied Protein Services, Lonza Biologics PLC, Cambridge, United Kingdom; 3 Antagonis Biotherapeutics GmbH, Graz, Austria

**Keywords:** chronic inflammatory, chemokines, CXCL8, glycosaminoglycans, drug candidate, *in silico* screening, immunogenicity

## Abstract

**Introduction:**

Chronic inflammatory processes are characterized by the infiltration of chemokine-activated leukocytes into inflamed tissues. Chemokines interact with their respective G protein-coupled receptors (GPCR) on target immune cells as well as with glycosaminoglycans (GAGs), displayed as part of proteoglycans, on the surface of endothelial cells to exert their function. Excessive levels of CXCL8 are associated with several chronic inflammatory diseases, which are characterized by a rich neutrophil influx. The CXCL8-GAG axis therefore represents a new therapeutic route to target these diseases.

**Methods:**

An anti-inflammatory CXCL8-based, dominant-negative mutant chemokine (termed dnCXCL8) with increased GAG binding affinity and knocked out GPCR activity, was generated and was proven to be active in various *in vivo* models in previous studies. To investigate the immunogenic potential of this dominant-negative mutant in comparison to its wild-type form, an *in vitro* T-cell activation study was performed.

**Results:**

Both proteins induced immunogenic responses, with dnCXCL8 showing a slightly higher response than wild-type CXCL8. An increased number of responsive donors for the mutant was detected, but no significant differences were observed at the population level. Concerning protein-derived peptides, the mutant-derived ones showed an enhanced frequency of lymphocyte activation; however, the differences were not significant on the population level.

**Discussion:**

This study provides first insights into the immunogenic potential of a chemokine-based drug candidate and paves the way for further optimization of chemokine-based therapy.

## 1 Introduction

Chemokines are a large class of small, mainly basic proteins with molecular weights ranging from 8–14 kDa (1). They play central roles in immune regulation, particularly in the coordination of leukocyte recruitment, activation, and migration during inflammatory and immune-mediated diseases (2). Structurally, all chemokines contain two or four conserved cysteine residues in their N-terminal regions that form characteristic disulfide bonds, which are required for their conformational stability and biological function (3). Based on the number and spacing of these cysteines, chemokines are divided into four families: CC, CXC, C, and CX_3_C, with the CC and CXC families representing the majority (4).

CXCL8, also known as interleukin-8 (IL-8), is a member of the CXC chemokine family and consists of 99 amino acids, including a 22-amino-acid signal peptide. After cleavage, two major isoforms (72 or 77 amino acids) are produced, with additional N-terminal processing yielding shorter variants. Importantly, chemotactic potency is inversely correlated with peptide length, with shorter isoforms exhibiting stronger activity (5–7). CXCL8 mediates multiple neutrophil functions, including chemotaxis, oxidative burst, granule enzyme release, and production of reactive oxygen species (8). These activities are initiated through binding to the G protein–coupled receptors (GPCRs) CXCR1 and CXCR2 with different affinities. CXCR1, initially described as CXCL8-selective, also recognizes CXCL6 as an agonist, whereas CXCR2 displays broader promiscuity, binding CXCL8 together with several other CXC chemokines (9, 10). Engagement of these receptors leads to neutrophil migration, angiogenesis, calcium mobilization, respiratory burst, and granule release, followed by receptor internalization, recycling, and resurfacing that sustain responsiveness (11, 12).

A key feature of chemokine biology is their interaction with glycosaminoglycans (GAGs), such as heparan sulfate and chondroitin sulfate. These negatively charged linear polysaccharides form proteoglycans upon attachment to core proteins and range in molecular weight from 10–100 kDa, dependent on the number of repeating disaccharide units (13). GAGs are classified into six types based on sugar composition, linkage geometry, and position (14). Their interactions with chemokines are mediated by electrostatic forces with positively charged amino acids, as well as hydrogen bonding and Van der Waals interactions (15). Sulfation at multiple positions (2-O, 3-O, 6-O, and N) further contributes to their high negative charge density (16). Chemokine–GAG interactions are essential for presentation, immobilization, and structural activation, but also for chemokine multimerization (e.g., CXCL8, CXCL4, CCL5), which enhances stability (17). CXCL8 is immobilized on GAGs and becomes thus locally concentrated and protected from sequestration and degradation. In this way, CXCL8 is immobilized and protected from degradation, allowing it to form molecular guideposts that direct neutrophil transmigration across the endothelium into inflamed tissues (7, 18, 19). While this process is critical for resolving infection, its dysregulation contributes to persistent or chronic inflammation.

Consistent with its functions, CXCL8 is a key modulator of leukocyte migration and angiogenesis in numerous pathological settings, including cancer, infectious diseases, pulmonary disorders, multiple sclerosis, rheumatoid arthritis, and other inflammatory conditions (9, 20–22). As a potent neutrophil chemoattractant, CXCL8 plays a central role in directing neutrophils rapidly to sites of infection and inflammation.

Given its pathological importance, CXCL8 has been targeted for therapeutic intervention. A dominant-negative CXCL8 mutant (dnCXCL8 = CXCL8 [Δ6 F17K F21K E70K N71K]) was engineered to completely inactivate GPCR-mediated signaling – by deleting the six N-terminal amino acids containing the ELR motif – while enhancing GAG binding affinity through specific amino acid substitutions in the GAG-binding domain (21, 23). As a result, dnCXCL8 can displace wild-type CXCL8 from endothelial GAG surfaces without activating neutrophils in the bloodstream (24). This approach has demonstrated potent anti-inflammatory effects by preventing neutrophil infiltration into chronically inflamed tissues. The anti-inflammatory and anti-migratory properties of dnCXCL8 have been validated in multiple *in vivo* models of acute and chronic inflammation, including models of cystic fibrosis (25, 26).

However, for biopharmaceuticals such as dnCXCL8, the intrinsic immunogenicity is a critical parameter that can influence both therapeutic efficacy and patient safety (27). After several fatal events, immunogenic assessment has become increasingly important. For example, a formulation change in erythropoietin alpha (Eprex^®^) provoked a severe immunological response, leading to pure red cell anaplasia in 250 patients with chronic renal failure. And even more severely, when in the TGN1412 (CD28-SuperMAB) trial a cytokine storm induction led to life-threatening events (28–31).

Based on these considerations, we investigated the potential of amino acid substitutions in dnCXCL8 to generate novel T-cell epitopes, thereby potentially altering its immunogenic profile compared to the wild-type protein. Since the display of drug-derived epitopes on MHC class II molecules of antigen-presenting cells is a prerequisite for initiating an immune response (30), we first employed an *in silico* prediction approach to identify potential CD4^+^ T-cell epitopes (32) in dnCXCL8 relative to wild-type CXCL8. The top-scoring predicted epitopes, together with the full-length proteins, were subsequently tested *in vitro* to determine their ability to elicit CD4^+^ T-cell responses through engagement of T-cell receptors (TCRs).

## 2 Materials and methods

This is a pure *in silico* and *in vitro* study, no animal experiments were undertaken.

### 2.1 *In silico* T-cell epitope profiling

The Epibase^®^ platform was used to predict and to screen for T-cell epitopes. This structure-based approach was capable of predicting peptide-MHC II interactions from various HLA allotypes of individuals of European, East Asian, Hispanic, and African American ancestry (33). The sequences of CXCL8 (Uni Prot ID P10145) and dnCXCL8 (**Table 1**) were scanned for the presence of putative HLA class II-restricted epitopes (T helper - epitopes) using the Epibase^®^ T-cell epitope profiling platform. With this platform, the HLA binding specificities of all possible 15-mers derived from the target sequences were analyzed. Profiling was done at the allotype level for 48 HLA class II receptors, namely 20 DRB1, 7 DRB3/4/5, 14 DQ, and 7 DP receptors (see **Supplementary Data 1**). The Epibase^®^ platform calculated a quantitative estimate of the free energy of binding ΔGbind of a peptide for each of the 48 HLA class II receptors. The free energies were then converted into dissociation constant (Kd) values through ΔGbind= RT ln(Kd). Peptides were classified as strong binders (Kd < 0.1µM), medium binders (0.1µM < Kd < 0.8µM) and non-binders (0.8µM < Kd) (33, 34).

**Table 1:** Amino acid sequences of wtCXCL8, dnCXCL8 (Δ6 F17K F21K E70K N71K), and CXCL8 derived peptides.

	10										20												30													
wtCXCL8	S	A	K	E	L	R	C	Q	C	I	K	T	Y	S	K	P	F	H	P	K	F	I	K	E	L	R	V	I	E	S	G	P	H	C	A	N
dnCXCL8	–	–	–	–	–	–	C	Q	C	I	K	T	Y	S	K	P	K	H	P	K	K	I	K	E	L	R	V	I	E	S	G	P	H	C	A	N
AP64							C	Q	C	I	K	T	Y	S	K	P	F	H	P	K	F	I	K	E												
AP65							C	Q	C	I	K	T	Y	S	K	P	K	H	P	K	K	I	K	E												
AP66												T	Y	S	K	P	F	H	P	K	F	I	K	E	L	R	V	I	E							
AP67												T	Y	S	K	P	K	H	P	K	K	I	K	E	L	R	V	I	E							

	40												50										60											70		
wtCXCL8	T	E	I	I	V	K	L	S	D	G	R	E	L	C	L	D	P	K	E	N	W	V	Q	R	V	V	E	K	F	L	K	R	A	E	N	S
dnCXCL8	T	E	I	I	V	K	L	S	D	G	R	E	L	C	L	D	P	K	E	N	W	V	Q	R	V	V	E	K	F	L	K	R	A	K	K	S
AP68																			E	N	W	V	Q	R	V	V	E	K	F	L	K	R	A	E	N	S
AP69																			E	N	W	V	Q	R	V	V	E	K	F	L	K	R	A	K	K	S

### 2.2 Proteins and peptides

CXCL8 and dnCXCL8 (Δ6, F17K F21K E70K N71K) were expressed in *E.coli* BL21 (Star™ DE3) and purified in three chromatographic steps: cation-exchange chromatography, reverse-phase C18 high-performance liquid chromatography (RP-HPLC), and a second cation-exchange step (23). The purified proteins were subsequently formulated by dialysis against phosphate-buffered saline (PBS). Endotoxin levels were determined using the Limulus Amebocyte Lysate (LAL) assay and complied with internal quality requirements of <0.06 EU/mL per batch. All peptides were custom-synthesized by Thermo Scientific at a purity level of >95 percent and delivered as trifluoroacetate (TFA) salts, representing the supplier’s standard formulation. Peptides (AP64, AP65, AP66, AP67, AP 68, AP69) were dissolved in DMSO, and all proteins/peptides were used at a 2.5 µg/ml concentration (corresponding to ~1.1–1.3 µM, depending on sequence-specific molecular weight). TFA at such high dilutions was not expected to raise any immunogenic-like effect. The amino acid sequences of all proteins and peptides are depicted in **Table 1**. The control peptides Tetanus toxoid (TT) were ordered from Statens Serum Institute and Keyhole Limpet Hemocyanin (KLH) from Calbiochem and used at 10 µg/mL concentration. AP3 (Kabat IGHG1), AP9 (Kabat IGHG1*011 CH2), AP70 (Pan DR sequence) were used at 2 µM and CEFT MHC II peptide mix at 1 µg/mL. Sequences of these peptides are depicted in **Table 2**.

**Table 2:** Predicted binding profile, source and sequence information of control peptides.

Peptide name	Predicted binding	Source	Sequence
AP3 (self)	Non-binding	Kabat IGHG1	APSSKSTSGGTAALG
AP9 (self)	promiscuous binding	Kabat IGHG1*01 CH2)	NSTYRVVSVLTVLHQ
AP70 (non-self)	promiscuous binding	Pan DR sequence	AKFVAAWTLKAAA
CEFT (non-self)		9 HLA restricted T-cell epitopes from tetanus, EBV, influenza	FVFTLTVPSER SGPLKAEIAQRLEDV DRLRRDQKS AGLTLSLLVICSYLFISRG TSLYNLRRGTALA AEGLRALLARSHVER PGPLRESIVCYFMVFLQTHI GQIGNDPNRDIL QYIKANSKFIGITEL

The predicted binding profile refers to the outcome of the Epibase^®^*in silico* HLA class II binding analysis, in which peptide–MHC II interactions were assessed across a panel of 48 representative HLA allotypes. *Non-binding* indicates that no relevant interactions were predicted at the chosen affinity threshold, while *promiscuous binding* indicates predicted binding to multiple HLA class II allotypes. Control peptides included self-derived sequences (AP3, AP9) and non-self sequences (AP70, CEFT).

### 2.3 PBMC preparation and stimulation

Peripheral blood mononuclear cells (PBMCs) were freshly prepared from whole blood obtained from healthy donors who had volunteered and provided informed consent. All blood donations were performed anonymously. Donors were screened according to internal criteria; however, no information regarding age, sex, or further demographics is available. Donor recruitment, informed consent, and ethical approval for the use of blood samples were managed by Algonomics in accordance with applicable regulatory standards (“No ethical approval is necessary as the study material is anonymous and voluntarily provided.” according to the Declaration of Helsinki). PBMCs were prepared from whole blood of healthy donors within 6 hours after withdrawal using Ficoll density-gradient centrifugation. The cells were cryopreserved in liquid nitrogen until use. A short-term polyclonal T-cell activation assay using anti-CD3 (0.03 μg/mL) was used to assess the quality of PBMC preparation. The Innogenetics InnoLIPA (Fujirebio Europe) HLA typing kit was used to determine DRB1 HLA allotypes at the 2-digit level for all donors. CD14+ cells were selected using the magnetic separation technique.

PBMCs were thawed and seeded at 1–3 × 10^5^ cells per well in AIM-V medium (Thermo Fisher Scientific) supplemented with IFN-γ (100 IU/mL) in round-bottom 96-well plates. After a 3 h incubation at 37 °C, antigen solutions were added (six donors per run). Cells were incubated for 7 days in a CO_2_ incubator at 37 °C. For re-stimulation a total of 1–4 × 10^4^ CD14^+^ cells were added per well together with fresh IFN-γ solution. Antigen solutions were re-applied after 3 h incubation at 37 °C. Two days after re-stimulation, cells were stained and analyzed on a FACS Canto II cytometer equipped with a High Throughput Sampler (HTS).

### 2.4 FACS analysis

Cell surface markers were stained using anti-CD3-APC-H7 (BD Biosciences), anti-CD4-Alexa488 (BD Biosciences), and 7-AAD (PerCP, BD Biosciences) for the selection of viable CD3^+^CD4^+^ cells. To evaluate activation, anti-CD25-PE (Miltenyi Biotec) and anti-CD137-APC (BD Biosciences) were used. All samples were analyzed on a FACS Canto II cytometer (BD Biosciences) equipped with a High Throughput Sampler (HTS).

Gating was performed in the first selection of CD3+CD4+ cells, and then the activated CD3+CD4+ cells were further selected for CD25+ or CD137 +. The latter cell population is used to set the threshold on the CD25 fluorescence that correlates with the activated status of the cells. The number of activated lymphocytes per well was calculated, normalizing the cell count to the number of beads added to each well. For each donor, three 96-well plates were processed. Each plate contained one blank control, 2–4 antigen conditions measured in 10 replicates, and two control antigens measured in 5 replicates. KLH was tested at 10 µg/mL. Sequence-related peptides were evaluated on the same plate.

### 2.5 Assessment of the immunogenicity profile

The comparison of T-cell activation in antigen-treated wells to untreated reference wells was used to assess intrinsic immunogenicity responses to an antigen. As the full-length protein usually has numerous epitopes, there’s a good chance to find an antigen-specific precursor T-cell in each of the 10-plicate analysis’ wells. As a result, for comparison of protein-treated wells to untreated wells, mean values of activated cells/well over 10-plicate wells (technical replicates) have been employed, resulting in a so-called stimulation index (SI). While the SI for whole-protein stimulation was assumed to follow a normal distribution - because multiple epitopes increase the likelihood of consistent responses across replicate wells - the responses to individual peptides were not assumed to be normally distributed, as peptide-specific precursor T cells are rare and replicate wells may contain either background or true stimulation values. A threshold value was set on the SI-value based on the stimulation index and the variation between replicates to determine if a donor/protein combination was positive or negative. Thus, the number of activated CD3+CD4+ T-lymphocytes in antigen-treated versus untreated wells was compared using SI-values.

Furthermore, comparing mutant protein-treated to wild-type protein-treated wells yields relative responses (RR), showing that both protein variations are immunogenic in a similar way. Positive control peptides of protein-induced immunogenicity, TT, and KLH were measured in 10-plicates to qualify the assay and statistical data interpretation. Peptide-mediated T-cell activation assays were qualified by the negative controls of a non-binding peptide AP3, and a predicted strong and promiscuous binding “self” peptide (germline IgG1) AP9. As positive controls, a mix of “recall” peptides from influenza, tetanus, and EBV (CEFT class II) and a “non-self” Pan DR sequence AP70 were incorporated in the peptide assay.

Standard statistical error tools were used (95% confidence interval) on the Δ-values between the mean values of the log (absolute numbers), which is related to the ratio (SI) of the absolute numbers. An identical procedure was used to calculate relative responses of mutant variants compared with wild-type proteins.

### 2.6 Statistical analysis

For each donor/protein combination, SI-values, 95% confidence intervals, and p-values were calculated. An antigen was assumed inducing an immunogenic response when the SI is ≥ 1.5. The criterion SI ≥1.5 supported by a p-value ≤0.05 to be listed as positive. Alternatively, significant decreases of immunogenic responses were considered when the SI value was ≤0.66, also supported by a p-value ≤0.05.

While the stimulation index (SI) for whole-protein stimulation was assumed to follow a normal distribution, the response to individual peptides was not. Depending on the underlying precursor frequencies, data from the 10-plicates of peptide-induced T-cell activation studies were not necessarily normally distributed, meaning some values were within the range of background, whereas others show stimulation. To address this, individual wells were classified as activated or not, and the frequency of positive wells was used for further statistical analysis. Linear discriminant analysis was performed to classify the status of each well automatically. This approach explored the discriminating power of each of the continuous variables separately and identified a set of response variables that showed a better discriminating power than each of the single variables. The list of variable parameters used in the automated classification tool was depicted in **Supplementary Data 2**.

To identify responsive donors for peptides, a pairwise comparison of proportions (Fisher’s Exact test) was performed for treatment and the untreated condition. Because peptide-specific precursor T cells are rare and responses may be sporadic across replicate wells, a less stringent threshold of p < 0.1 was applied in this exploratory screening assay to minimize false negatives. A donor was considered responsive when the frequency of the peptide treated condition was different from the untreated condition with a p-value <0.1.

At the population level, peptide-induced immunogenicity was analyzed using a linear mixed model with a fixed part (differences between conditions) and a random part accounting for donor and plate effects. Peptide averages were estimated in the presence of random effects for donor and plate, and all estimates of the peptide’s effects were computed within plate and donor. This statistical analysis allowed an assessment of the impact of the peptide treatments on the mean frequency of activated wells at the population level over all donors.

To evaluate the relative immunogenicity of mutant peptides compared with their wild-type counterparts, the wild-type peptide condition was taken as a reference level. This enabled investigation of the impact of site-specific mutations on peptide immunogenicity. The applied criteria correctly ranked the positive and negative control peptides and confirmed the expected frequency of positive donors for the control peptides.

## 3 Results

### 3.1 *In silico* screening

Only peptides that bind with a sufficiently high affinity to HLA class II receptors can be presented on the cell surface to initiate a T-helper (TH) response (30, 35). Therefore, determining peptides within a protein sequence that has a strong affinity for HLA class II receptors was the first step in assessing the immunogenic potential of CXCL8 and dnCXCL8. This was done using the Epibase^®^ platform, which is based on a structure-based method taking into account the 3D structure of the binding groove of the HLA II molecules. DRA/DRB1 was in the primary focus of immunogenicity profiling, as its expression level is the highest (33, 34, 36).

Our *in silico* screening showed that both analyzed proteins contained moderate amounts of TH epitopes specific for a significant part of the population. These epitopes were located in two allocated clusters of the two molecules. The results provided the first prediction of potential immunogenic epitopes based on the primary amino acid sequence of these proteins. This suggests that some of these epitopes might be immunogenic upon high dosing or repeated administration of the proteins. However, factors such as formulation of the product, aggregation, or post-translational modifications could not be considered in this *in silico* approach. Therefore, peptides for *in vitro* T-cell activation studies were designed based on the results obtained from the *in silico* study (**Table 3**) and were then investigated in more detail *in vitro.*

**Table 3:** Summary of *in silico* TH profiling of wtCXCL8 and dnCXCL8.

	DRB1		DRB3/4/5		DQ		DP	
	Strong	Medium	Strong	Medium	Strong	Medium	Strong	Medium
CXCL8	5	9	1	2	0	6	1	3
dnCXCL8	4	13	3	3	0	5	0	2

Peptides binding to multiple HLAs of the same group (DRB1, DRB3/4/5, DP, DQ) were counted as one. Predicted binding strength was classified by Epibase^®^ according to Kd values: strong (<0.1 μM) or medium (0.1–0.8 μM).

The summary of the *in silico* TH profiling of CXCL8 and dnCXCL8 is displayed in **Table 3**. The majority of binders were found for DRB1, which is in accordance with experimental evidence that allotypes belonging to the DRB1 are more potent peptide binders (37). CXCL8 contains 5 strong DRB1 binders, whereas dnCXCL8 contains 4 strong DRB1 binders. No promiscuity in strong DRB1 binders was observed. In addition, 9 medium-strength DRB1 binders were found within the CXCL8 sequence and 13 such peptides within dnCXCL8. CXCL8 contains 1 strong binder for DRB3/4/5, 1 for DP, and none for DQ. Respectively 2, 6, and 3 medium binders to DRB3/4/5, DQ, and DP were found. dnCXCL8 contains 3 strong binders for DRB3/4/5 and none for DP or DQR. Respectively 3, 5, and 2 medium binders to DRB3/4/5, DQ, and DP were found (**Table 3**). The allotype with the highest apparent immunogenic risk for CXCL8 was DRB1*0402 with 2 strong binders; the one for dnCXCL8 is DRB5*0101 with 3 strong binders (**Supplementary Data 3**). In both proteins, the potentially immunogenic epitopes were not distributed randomly but were concentrated in two discrete clusters along the amino acid sequence (**Table 4**).

**Table 4:** Mapping of Epibase^®^ predictions in the classical 15-mer peptide format.

Start	CXCL8			dnCXCL8		
	15-mer sequence	Allotype count	Implicated serotypes	15-mer sequence	Allotype count	Implicated serotypes
1	SAKELRCQCIKTYSK	0				
4	ELRCQCIKTYSKPFH	0				
7	**CQCIKTYSKPFHPKF**	5	DR11 (5), DR14(6), DR4, DR8	**CQCIKTYSKPKHPKK**	5	DR11(5), DR14(6), DR4, DR8
10	**IKTYSKPFHPKFIKE**	5	DR11(5), DR14(6), DR4, DR8	**IKTYSKPKHPKK IKE**	6	DR11(5), DR14(6), DR15(2), DR4, DR8
13	**YSKPFHPKFIKELRV**	4	-, DPw2, DR4, DR8	**YSKPK HPKK IKELRV**	3	DR15(2), DR4, DR8
16	**PFHPKFIKELRVIES**	4	-, DPw2, DR4, DR8	**PK HPKK IKELRVIES**	2	DR4, DR8
19	PKFIKELRVIESGPH	2	DR1, DR4	**PKK IKELRVIESGPH**	2	DR1, DR4
22	IKELRVIESGPHCAN	5	DR1, DR13(6), DR4	IKELRVIESGPHCAN	5	DR1, DR13(6), DR4
25	LRVIESGPHCANTEI	4	DR1, DR13(6)	LRVIESGPHCANTEI	4	DR1, DR13(6)
28	IESGPHCANTEIIVK	0		IESGPHCANTEIIVK	0	
31	GPHCANTEIIVKLSD	0		GPHCANTEIIVKLSD	0	
34	CANTEIIVKLSDGRE	0		CANTEIIVKLSDGRE	0	
37	TEIIVKLSDGRELCL	0		TEIIVKLSDGRELCL	0	
40	IVKLSDGRELCLDPK	0		IVKLSDGRELCLDPK	0	
43	LSDGRELCLDPKENW	0		LSDGRELCLDPKENW	0	
46	**GRELCLDPKENWVQR**	3	DR13(6), DR15(2), DR17(3)	**GRELCLDPKENWVQR**	3	DR13(6), DR15(2), DR17(3)
49	**LCLDPKENWVQRVVE**	4	DR13(6), DR15(2), DR17(3), DR4	**LCLDPKENWVQRVVE**	4	DR13(6), DR15(2), DR17(3), DR4
52	**DPKENWVQRVVEKFL**	1	DR4	**DPKENWVQRVVEKFL**	1	DR4
55	**ENWVQRVVEKFLKRA**	5	DR11(5), DR13(6), DR15(2), DR51	**ENWVQRVVEKFLKRA**	5	DR11(5), DR13(6), DR15(2), DR51
58	**VQRVVEKFLKRAENS**	6	DR11(5), DR13(6), DR15(2), DR51, DR8	**VQRVVEKFLKRAKK S**	7	DR11(5), DR13(6), DR15(2), DR4, DR51, DR8

This table shows the allotype count of critical epitopes and implicated serotypes for each 15-mers spanning the CXCL8 and dnCXCL8 sequences. The column “Start” indicates the position of the first amino acid of each 15-mer relative to the mature protein sequence. Predicted epitopes are highlighted in bold to indicate clusters of potentially immunogenic regions, which were identified in both proteins. These clusters correspond to consecutive peptides with multiple predicted binders, suggesting localized regions of higher immunogenic potential.

In both proteins, the predicted immunogenic epitopes were not evenly distributed but instead concentrated in two discrete regions of the sequence. The first cluster was located in the N-terminal region, spanning residues 7–22, where consecutive 15-mers showed binding to multiple HLA class II serotypes (4–6 implicated serotypes each in both CXCL8 and dnCXCL8). The second cluster was located in the C-terminal region, spanning residues 49–58, again comprising consecutive peptides with high allotype counts (4–7 implicated serotypes). These clusters represent localized hot-spots of potential immunogenicity (**Table 4** and **Supplementary Data 3**).

### 3.2 Protein *in vitro* immunogenicity

Protein *in vitro* immunogenicity responses were defined as an increase in the average number of activated (CD25 high) CD4+ cells per well for antigen-treated wells compared to untreated blank wells (contrast), expressed as stimulation index (SI). The SI-value with 95% confidence interval and p-value (SI = 1) was calculated for each antigen or reference. The results for the control antigens KLH and TT, as well as for CXCL8 and dnCXCL8, are summarized in **Table 5**, with the complete dataset including 95% CI available in **Supplementary Data 4**, **5**.

**Table 5:** Summary table of antigen-induced intrinsic and relative immunogenic responses.

Donor	Keyhole Limpet Hemocyanin		Tetanus Toxoid		wtCXCL8		dnCXCL8	
	SI	P-value	SI	P-value	SI	P-value	SI	P-value
AIV00008	1,16	0,665	2,34	0,007	1,16	0,559	1,01	0,979
AIV00011	2,04	0,000	2,46	0,000	0,98	0,869	0,86	0,254
AIV00020	1,27	0,025	1,47	0,000	1,07	0,432	0,90	0,208
AIV00021	2,43	0,006	8,76	0,000	1,67	0,051	1,05	0,853
AIV00026	3,53	0,001	21,87	0,000	1,41	0,256	2,67	0,001
AIV00032	1,41	0,059	2,40	0,000	1,09	0,548	1,20	0,217
AIV00036	3,55	0,000	18,71	0,000	1,40	0,172	5,50	0,000
AIV00038	2,14	0,015	7,35	0,000	1,05	0,867	1,25	0,383
AIV00039	4,91	0,002	21,46	0,000	4,20	0,000	5,05	0,000
AIV00041	3,10	0,001	26,69	0,000	2,65	0,001	2,66	0,000
AIV00049	1,89	0,054	13,44	0,000	1,15	0,604	5,60	0,000
AIV00054	2,43	0,011	6,74	0,000	2,08	0,01	1,85	0,048
AIV00059	2,93	0,000	8,63	0,000	2,08	0,000	4,67	0,000
AIV00062	2,63	0,000	6,09	0,000	2,83	0,000	2,72	0,000
AIV00068	2,43	0,000	1,20	0,341	1,41	0,03	0,89	0,459
AIV00070	2,30	0,021	2,02	0,052	1,61	0,103	1,33	0,336
AIV00071	1,89	0,000	6,25	0,000	1,36	0,028	0,72	0,02
AIV00072	1,27	0,079	3,63	0,000	1,99	0,000	1,70	0,000
AIV00076	3,31	0,001	4,66	0,000	2,22	0,008	1,94	0,038
AIV00079	1,14	0,343	1,11	0,431	1,29	0,022	0,77	0,017
AIV00082	1,38	0,193	4,86	0,000	1,00	0,997	1,24	0,286
AIV00084	0,98	0,938	1,46	0,099	0,80	0,221	0,54	0,001
AIV00091	2,62	0,000	2,03	0,005	1,67	0,017	1,75	0,008
AIV00104	1,77	0,046	3,75	0,000	1,36	0,183	0,91	0,691
AIV00108	2,73	0,004	5,38	0,000	4,31	0,000	1,80	0,036
AIV00118	4,29	0,000	21,43	0,000	7,61	0,000	5,36	0,000
AIV00123	1,55	0,005	1,56	0,005	1,29	0,046	1,04	0,733
AIV00129	4,24	0,000	9,02	0,000	1,49	0,227	1,57	0,172
AIV00130	1,87	0,025	2,53	0,001	0,96	0,841	1,95	0,004
AIV00135	2,59	0,006	4,29	0,000	1,26	0,409	2,26	0,004
AIV00137	4,24	0,000	0,93	0,845	1,92	0,025	1,86	0,032
AIV00138	3,10	0,017	2,29	0,079	2,52	0,017	2,51	0,018
AIV00144	1,23	0,318	1,27	0,244	1,02	0,925	0,84	0,318
AIV00153	3,00	0,000	2,54	0,001	2,23	0,000	1,36	0,162
AIV00159	1,57	0,000	3,90	0,000	0,91	0,426	0,88	0,181
AIV00160	1,99	0,000	2,91	0,000	1,10	0,466	1,09	0,518
AIV00162	1,55	0,02	2,26	0,000	1,29	0,076	1,33	0,046
AIV00165	3,08	0,005	29,30	0,000	2,35	0,01	1,54	0,184
AIV00166	2,18	0,01	5,83	0,000	0,84	0,509	4,00	0,000
AIV00167	2,53	0,000	4,64	0,000	1,19	0,273	1,22	0,166
AIV00171	1,47	0,249	6,94	0,000	0,86	0,583	6,11	0,000
AIV00172	1,88	0,000	1,71	0,000	1,04	0,737	1,52	0,001
AIV00173	1,04	0,883	8,38	0,000	0,72	0,116	2,51	0,000
AIV00174	0,98	0,962	1,91	0,037	0,65	0,094	0,87	0,58
AIV00175	1,29	0,107	1,81	0,000	0,91	0,47	1,01	0,926
AIV00176	1,12	0,601	0,96	0,841	1,07	0,684	0,72	0,047
AIV00178	1,65	0,134	4,60	0,000	1,14	0,641	1,09	0,751
AIV00179	2,36	0,003	13,21	0,000	1,42	0,14	1,38	0,179
AIV00180	3,24	0,008	2,01	0,112	1,08	0,827	1,00	0,995
AIV00181	2,76	0,013	2,23	0,049	1,83	0,07	1,18	0,61
AIV00182	2,96	0,000	5,44	0,000	1,35	0,184	2,01	0,002
% responsive donors	71		80		27		43	

In the intrinsic immunogenicity table, SI values of all donor/protein combinations compared with the corresponding blank condition are given. Light grey boxes indicate the antigen induces immunogenic responses (SI>1.50 and p-value (SI = 1)<0.05). Dark grey boxes indicate donor/protein combinations that result in a significantly reduced immunogenicity (SI<0.66 and p-value (SI = 1)<0.05).

An immunogenic response is defined as SI≥1.5 supported by a p-value (SI = 1) ≤0.05, whereas a significant reduction is defined as SI value ≤0.66 supported by a p-value (SI = 1) ≤0.05. The defined threshold was set to RR > 1,5 supported by p-value (RR = 1) ≤0.05 or RR<0.66 supported by p-value (RR = 1) <0.05 with the inclusion that at least for 1 of the proteins a significant enhanced or reduced SI for that donor was observed. RRs were calculated for each donor/contrast combination to compare mutant dnCXCL8 with wild-type CXCL8. For each RR value, 95% confidence intervals and p-values (RR = 1) were determined. This analysis indicates whether the immunogenic responses elicited by the mutant protein differ significantly from those of the wild-type. The results are summarized in **Table 6**, where a mean RR = 1 reflects similar immunogenicity of dnCXCL8 and CXCL8.

**Table 6:** The relative immunogenicity table indicates the relative responses of dnCXCL8 with wtCXCL8 per donor.

Donor	dnCXCL8 vs. CXCL8	AIV00118	0,7
AIV00008	0,87	AIV00123	0,81
AIV00011	0,88	AIV00129	1,05
AIV00020	0,84	AIV00130	2,04
AIV00021	0,63	AIV00135	1,79
AIV00026	1,9	AIV00137	0,97
AIV00032	1,1	AIV00138	0,99
AIV00036	3,93	AIV00144	0,83
AIV00038	1,19	AIV00153	0,61
AIV00039	1,2	AIV00159	0,97
AIV00041	1	AIV00160	0,99
AIV00049	4,87	AIV00162	1,03
AIV00054	0,89	AIV00165	0,66
AIV00059	2,25	AIV00166	4,75
AIV00062	0,96	AIV00167	1,03
AIV00068	0,63	AIV00171	7,09
AIV00070	0,82	AIV00172	1,46
AIV00071	0,53	AIV00173	3,49
AIV00072	0,86	AIV00174	1,34
AIV00076	0,87	AIV00175	1,11
AIV00079	0,6	AIV00176	0,68
AIV00082	1,24	AIV00178	0,96
AIV00084	0,68	AIV00179	0,97
AIV00091	1,04	AIV00180	0,92
AIV00104	0,67	AIV00181	0,65
AIV00108	0,42	AIV00182	1,49

Colored boxes indicate the protein variant induces significantly different responses than the wild-type protein: grey colors encode significantly higher immunogenic responses induced by the variant protein. Dark grey indicates higher immunogenic responses of the wild-type protein. Thresholds for these assignments are RR>1.5 or RR<0.66, respectively, and p-value (RR = 1)<0.05), involving at least for 1 of the proteins a significant SI for that donor.

To exclude endotoxin-related artifacts, all recombinant protein batches were tested for endotoxin content using the Limulus Amebocyte Lysate (LAL) assay, with values confirmed to be below of an internal acceptance criterion of 0.06 EU/mL per batch.

**Figure 1:**
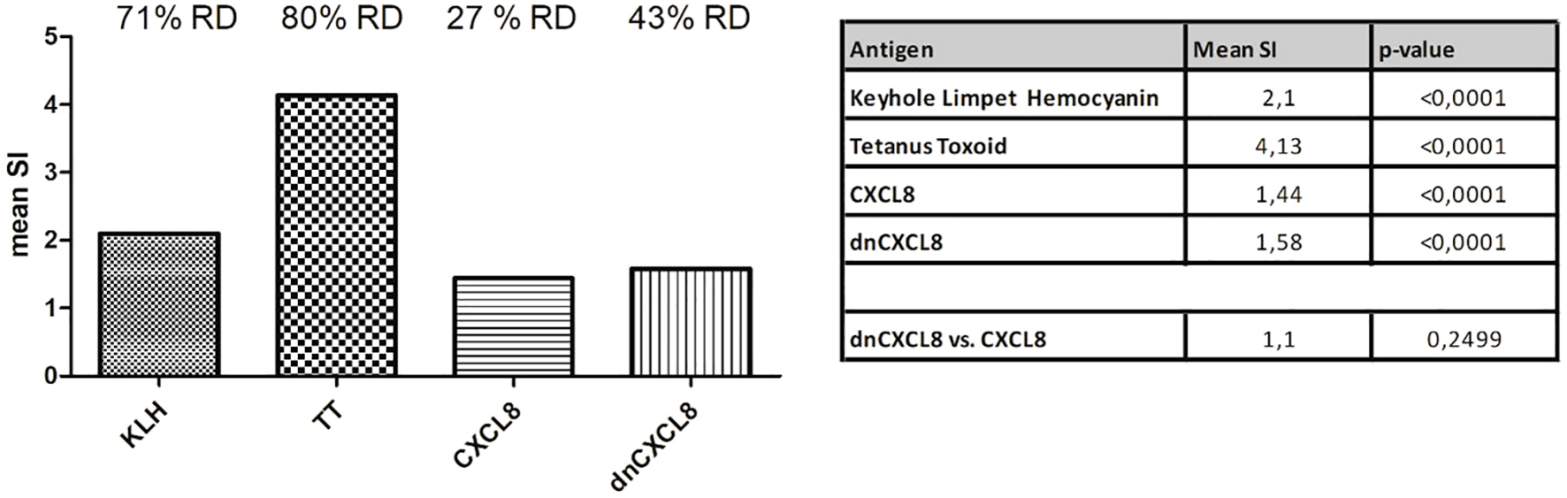
Mean intrinsic immunogenicity data for CXCL8 and dnCXCL8 compared to control antigens in a 51-donor population. Left: Bar graph representation of the mean stimulation index (SI) for Keyhole Limpet Hemocyanin (KLH), Tetanus Toxoid (TT), CXCL8, and dnCXCL8, together with the percentage of responsive donors (RD). Right: Tabular representation of mean SI values and corresponding p-values for the same antigens.

**Figure 1** summarizes the mean immunogenicity responses (SI) in a 51-donor population for all tested samples. TT and KLH, which both served as positive control antigens, showed an evident 71% and 80% immunogenic potency. Both antigens showed a clear shift in a frequency distribution, which was consistent with the single donor level. The mean SI of KLH was 2.1, and of TT 4.13 in the population, both with p-values (SI = 1) less than 0.0001 (**Figure 1**). The mean relative immunogenicity responses for all 51 donors could be determined by comparing the population level immunogenicity of dnCXCL8 to CXCL8. **Figure 1** shows the mean RR values for protein variations and the associated p-value (RR = 1). The relative immunogenicity responses of KLH, TT, CXCL8, and dnCXCL8 compared to Blank, observed in all donors, is depicted in **Supplementary Data 6**-**9**. When assessing the whole donor SI distribution, the protein-mediated immunogenicity assays’ findings showed that all proteins are significantly immunogenic (p<0.05).

At the population level, dnCXCL8 displayed slightly higher immunogenicity than wild-type CXCL8, with mean SI values of 1.58 and 1.44, respectively. An increased number of responsive donors was obtained there, with 12 of the 14 CXCL8 responsive donors being positive for dnCXCL8. In ten of these donors, the response to the mutant protein was the same as it was to the wild-type protein. This suggests that T-cell activation in these donors was likely driven by epitopes present in the common regions of both proteins. In 9 donors, the mutant proteins had significantly higher relative responses than the wild-type proteins (RR>1.5 and p-value (RR = 1)<0.05), with only one of these donors being also responsive to CXCL8. But this difference in immunogenicity was not significantly different between dnCXCL8 and CXCL8. Donors with common characteristics may be more predisposed to respond significantly different to both proteins. Three donors showed a reduction in immunogenic response for dnCXCL8 compared to CXCL8. Only one donor sample, labeled as AIV00084, showed a significant decrease in T-cell activation, visible as reduced SI value. Still, in this sample, none of the control peptides induced a significant immunogenic response. Relative responses (RR) are calculated by comparison of the mean number of activated, CD25 high, CD4+ lymphocytes/well in CXCL8 versus dnCXCL8 samples for each donor. These RR values are summarized in **Table 6**, where a mean RR of 1 reflects comparable immunogenicity of mutant and wild-type proteins.

### 3.3 The peptides’ *in vitro* immunogenicity

**Figure 2:**
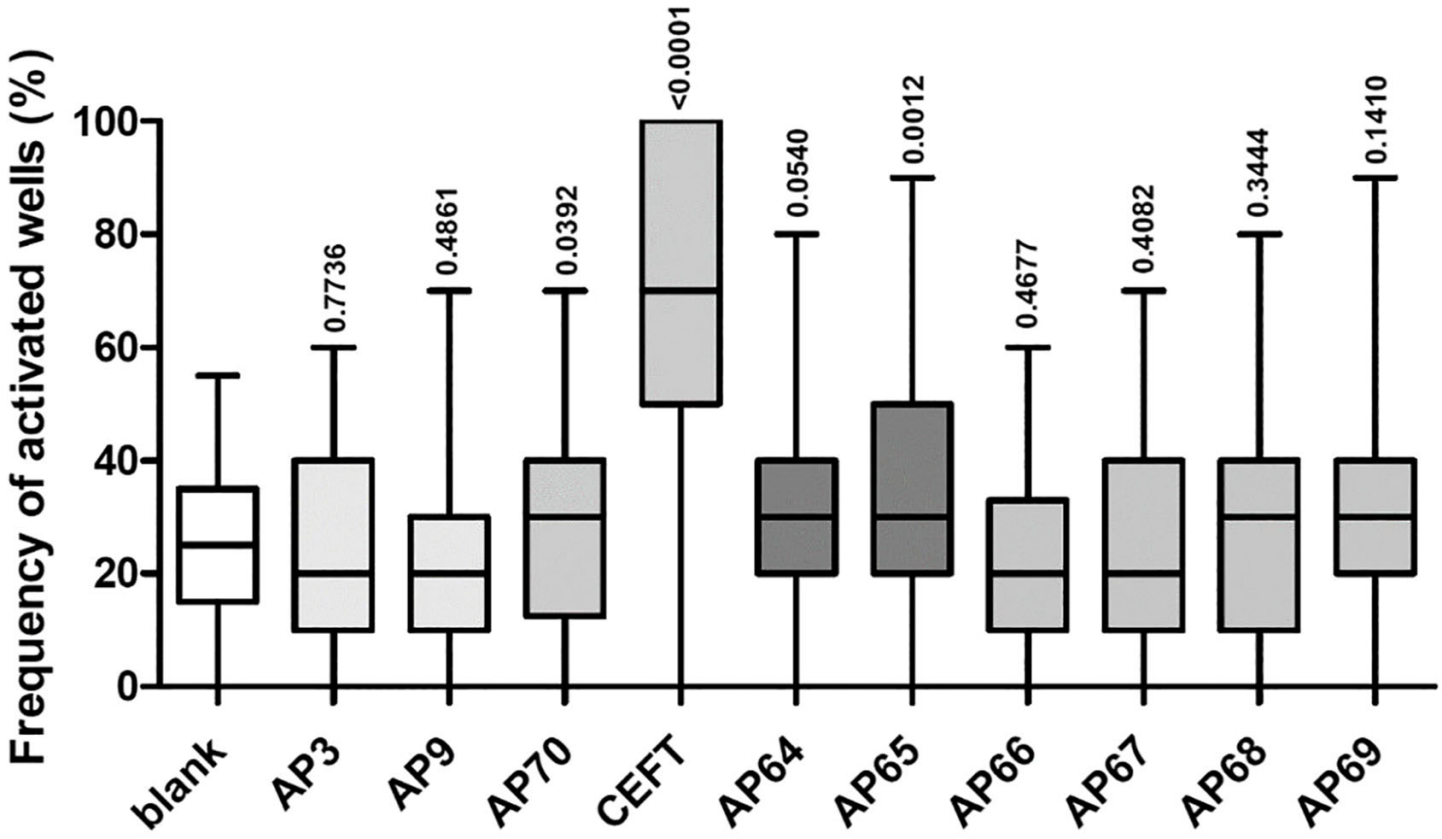
The frequency of activated wells for control peptides in a 51-donor population. Box-and-whisker plots showing the frequency of activated wells (%) for blank, control peptides (AP3, AP9, AP70, CEFT), and CXCL8/dnCXCL8-derived peptides (AP64–AP69). Median, interquartile range, and full range of values are displayed. The p-values shown above the plots indicate the statistical comparison between peptide-treated and untreated (blank) conditions.

Pairwise comparison of proportions of frequency of activated wells in the peptide-treated condition and the untreated condition was performed (**Table 5**). The untreated control (blank) depicted greater values than 0, which is attributed to donor-dependent random, non-specific activation in the absence of exogenous antigen that is presented to the T-cells. When compared to “untreated” activation, there was a lower overall activation level in the presence of peptides in the assay. In most cases, the decrease in frequency is not statistically significant, however for some peptide/donor combinations, the negative difference (decrease) in frequency is statistically significant with p<0.1 or even p<0.05. This categorization into responsive and non-responsive donors rejects borderline responses. A graphical representation of the range of activation frequencies across donors is provided in **Figure 2**.

The negative control peptides AP3 and AP9 induced minimal responses, with <2% of donors responding. The positive control peptides showed higher responsiveness: AP70 (Pan-DR) induced activation in ~16% of donors, while the CEFT peptide pool triggered responses in >60% of donors.

Strong immunogenic responses at the population level were detected for 1 of the 3 CXCL8-derived self-peptides, which corresponded to the significant immunogenicity observed for CXCL8. Even though the number of responsive donors was less than 6%, there is a considerable rise in the average frequency of activated wells in the population, indicating the contribution of donor samples with immunogenic reactions at the detection threshold. Although the wild-type based peptide AP68 induced responses in almost 10% of donors, the amplitude of the response was not drastically affecting the average frequency of activated wells in the total population (**Table 6**). The average frequency of activated wells was stronger for the non-self-peptides from dnCXCL8 compared to CXCL8. AP65 showed the highest immunogenic potency with almost 12% of positive donors. This peptide was found in the same N-terminal region as AP64 but with the two phenylalanine to lysine alterations present only in dnCXCL8. However, comparing the relative immunogenicity of AP65 and AP64 at the population level showed no substantial difference between the two peptides.

Furthermore, no significantly different immunogenicity was observed between mutated peptides derived from dnCXCL8 and their wild-type counterparts on the population level. For the peptides AP66 and AP67, only a limited number of donors showed a statistically significant decrease. However, other donor samples contributed to the negative difference in average frequency at the population level, resulting in this overall lower activation status (respectively 7 and 11 donors, a decrease of percent activated wells of more than 20 percent was observed). However, this diminished immunogenic response could not be classified as significant at the population level (**Table 7**). The observation was similar to the effect of the negative control peptide AP9.

**Table 7:** List of responsive donors for each peptide/donor combination.

	Blank	AP3	AP9	AP70	CEFT	AP64	AP65	AP66	AP67	AP68	AP69
AIV00008	20	0	11	30	75	10	10	50	10	0	20
AIV00011	44	50	50	60	100	30	70	60	60	33	40
AIV00020	16	10	10	70	100	40	20	20	20	33	10
AIV00021	29	10	10	10	100	30	40	30	10	20	40
AIV00026	0	0	29	0	11	40	0	0	0	0	0
AIV00032	30	50	60	60	100	56	40	33	44	50	60
AIV00036	30	10	10	20	100	40	20	30	50	10	20
AIV00038	15	30	33	70	60	50	50	50	50	50	30
AIV00039	0	20	11	50	100	10	60	10	0	20	20
AIV00041	45	40	22	40	60	80	20	30	40	20	40
AIV00049	15	30	0	12	67	33	20	0	0	10	0
AIV00054	35	60	40	30	30	70	70	20	30	80	70
AIV00059	32	20	30	40	90	30	80	40	10	60	44
AIV00062	40	20	20	20	50	70	10	20	20	30	40
AIV00068	20	20	10	30	100	30	40	10	40	30	30
AIV00070	40	20	20	10	100	20	0	20	0	10	20
AIV00071	55	10	0	10	50	20	10	20	20	20	20
AIV00072	30	20	10	70	30	50	90	0	44	0	50
AIV00076	35	40	60	30	40	70	67	50	50	60	40
AIV00079	5	0	10	0	29	0	0	0	0	0	0
AIV00082	11	40	20	60	100	40	30	40	30	30	70
AIV00084	40	44	11	20	100	50	20	60	20	20	0
AIV00091	20	50	10	44	100	10	60	30	10	40	30
AIV00104	20	20	0	20	90	40	20	30	20	20	0
AIV00108	25	30	10	50	60	20	20	0	20	20	20
AIV00118	5	20	20	0	0	20	30	30	10	10	10
AIV00123	40	30	40	22	100	30	40	30	10	40	30
AIV00129	25	10	10	10	70	30	0	20	20	20	20
AIV00130	25	30	10	40	100	40	80	20	0	60	30
AIV00135	15	20	20	40	70	22	56	10	40	20	20
AIV00137	40	50	70	20	90	30	50	40	70	40	50
AIV00138	15	0	20	20	70	80	40	20	10	40	20
AIV00144	35	30	30	70	40	60	80	40	40	70	40
AIV00153	35	10	10	40	0	20	50	10	38	30	20
AIV00159	5	0	11	10	20	10	20	0	0	0	30
AIV00160	40	33	30	30	100	25	50	40	30	20	30
AIV00162	50	50	50	30	100	30	20	50	50	33	60
AIV00165	5	20	20	10	50	10	20	0	0	40	10
AIV00166	15	30	30	40	50	0	30	20	30	40	10
AIV00167	20	40	50	60	100	50	70	20	50	60	70
AIV00171	15	10	10	40	100	0	10	0	0	33	30
AIV00172	53	40	30	40	100	40	70	10	10	20	70
AIV00173	5	0	0	0	11	0	0	0	10	33	10
AIV00174	20	10	10	10	10	25	30	30	0	10	20
AIV00175	25	40	20	30	50	40	10	10	30	50	50
AIV00176	40	40	60	40	50	0	50	40	10	30	90
AIV00178	0	20	10	10	30	10	20	10	10	10	0
AIV00179	10	20	10	30	100	20	40	20	10	10	20
AIV00180	30	30	50	40	100	30	10	30	50	40	20
AIV00181	35	10	30	20	60	50	50	10	20	20	40
AIV00182	30	0	20	11	70	0	40	0	0	0	20
# responsive donors	0	0	1	8	32	3	6	0	1	5	2
Average	24,7	23,8	22,5	30,3	68,3	31,6	35,9	22,8	22,5	28,3	30,1
	positive response with p<0,05.										
	positive response with 0,05 ≤ p ≤ 0,10.										
	negative response with 0,05 ≤ p ≤ 0,10.					
	negative response with p<0,05.					

A matrix depiction of the frequency of activated wells for each donor/peptide combination is displayed. Compared to untreated blank wells, the color-coding shows peptide/donor combinations that exhibit a significantly different frequency of activated wells. Peptide/donor combinations that exhibit a considerably greater frequency of activated wells than blank are labeled grey (0,05 ≤ p ≤ 0,10) and dark grey (p<0.05) in the comparison. Light grey (0,05 ≤ p ≤ 0,10) and black (p<0.05) are the colors for peptide/donor combinations with a significantly lower activation state.

The frequency of peptide-induced T-cell activation across donors was further analyzed. Several peptides elicited significantly higher activation frequencies compared with blank conditions (p < 0.1) and were therefore classified as immunogenic (**Table 8**). These results complement the stimulation index data by identifying specific peptide sequences that contributed most strongly to the observed immunogenic responses.

**Table 8:** Immunogenicity of peptides using flow cytometry in a 51-donor population.

	**Peptide ID**	**Sequence**	**%responsive donors** **(p<0,1)**	**immunogenic potential** **Δ% activated wells**	**P-value**
Negative Control	AP3	APSSKSTSGGTAALG	0,0	-0,951	0,7736
	AP7	NSTYRVVSVLTVLHQ	2,0	-2,304	0,4861
Positive Control	AP70	AKFVAAWTLKAAA	15,7	5,578	0,0392
	CEFT	FVFTLTVPSER SGPLKAEIAQRLEDV DRLRRDQKS AGLTLSLLVICSYLFISRG TSLYNLRRGTALA AEGLRALLARSHVER PGPLRESIVCYFMVFLQTHI GQIGNDPNRDIL QYIKANSKFIGITEL	62,7	43,108	<0,0001
CXCL8 Peptides	AP64	CQCIKTYSKPFHPKFIKE	5,9	6,382	0,054
	AP66	TYSKPFHPKFIKELRVIE	0,0	-2,402	0,4677
	AP68	ENWVQRVVEKFLKRAENS	9,8	3,127	0,3444
dnCXCL8 Peptides	AP65	CQCIKTYSKPK HPKK IKE	11,7	10,735	0,0012
	AP67	TYSKPK HPKK IKELRVIE	2,0	-2,735	0,4082
	AP69	ENWVQRVVEKFLKRAKK S	4,0	4,873	0,141

The difference between the frequency of activated wells in the peptide treated and blank conditions was assessed using pairwise comparison of proportions. The immunogenic potential of a peptide was expressed as the average difference in frequency of activated wells in the donor population. It was analyzed using a linear mixed model with the difference between treatment conditions as the fixed part of the model and the random part being plate and donor. Peptides with considerably greater frequency of activated wells (p<0.1) in the population than the blank condition were considered immunogenic. Amino acid changes in respect to wild-type protein are underlined.

To compare dnCXCL8 and wild-type CXCL8 directly, relative peptide responses were calculated on a donor-by-donor basis. Most peptides exhibited similar activation frequencies between the two proteins, while selected peptides displayed modest differences in immunogenic potential (**Table 9**). These relative comparisons provide additional evidence for the largely comparable immunogenicity profiles of dnCXCL8 and CXCL8.

**Table 9:** The relative immunogenicity of three pairs of peptides was assessed using flow cytometry in a 51 healthy donor samples.

	Average frequency (Δ%)	P-value
AP64 vs. AP65	4,353	0,2659
AP66 vs. AP67	-0,333	0,8991
AP68 vs. AP69	1,745	0,5599

The difference between the frequency of activated wells in the mutant and wild-type peptide treatment conditions was assessed using a pairwise comparison of proportions. The average difference in frequency of activated peptides was used to calculate a mutant peptide’s relative immunogenic potential. It was analyzed using a linear mixed model with the difference between treatment conditions as the fixed part of the model and the random part being plate and donor. A mutant peptide was considered to be significantly more immunogenic than the wild-type peptide if a significantly higher frequency of activated wells (p<0.1) for the mutant peptide compared with the wild-type peptide condition over the population was observed.

## 4 Discussion

Intrinsic immunogenicity is a critical parameter that directly impacts the therapeutic efficacy and safety of biopharmaceuticals. In this study, we investigated the immunogenic potential of a chemokine-based dominant-negative mutant, dnCXCL8, in comparison to its wild-type counterpart CXCL8, using both full-length proteins and peptide-derived epitopes. A panel of positive and negative control proteins and peptides, including Keyhole Limpet Hemocyanin (KLH), Tetanus toxoid (TT), AP3, AP9, CEFT, and AP70, confirmed the validity of the experimental approach and provided reference for interpretation of the responses.

### 4.1 Protein immunogenicity

Both CXCL8 and dnCXCL8 elicited measurable T-cell activation *in vitro.* While dnCXCL8 tended to induce slightly stronger responses than wild-type CXCL8, this difference was not statistically significant at the population level. This suggests that dnCXCL8 does not possess an intrinsically higher immunogenic potential compared with its progenitor molecule.

The detection of immunogenic responses against wild-type CXCL8 may initially appear unexpected, as tolerance to self-proteins would normally be anticipated. However, this finding is consistent with reports of anti-chemokine autoantibodies in healthy individuals, including antibodies against CXCL8, IL-1, IL-2, TNFα, IFNγ, IL-6, and CCL2 (38, 39). These autoantibodies may circulate as immune complexes or as free immunoglobulins (IgG, IgM, IgA), and their concentrations can increase under inflammatory conditions such as gastritis or rheumatoid arthritis (40, 41). Moreover, the assay conditions employed here differ from physiological settings, as supraphysiological protein concentrations (µM range vs. nM–pM *in vivo*) were used, which may amplify detectable responses (5). Additional factors such as protein purity, formulation, aggregation, and glycosylation status could also contribute to the observed immunogenicity (42). Taken together, these considerations provide a rationale for why immunogenic responses were observed even for wild-type CXCL8.

### 4.2 Peptide immunogenicity

At the peptide level, only two epitopes (AP64 and AP65) reached significance at the population level. These two peptides share the same sequence but are derived from CXCL8 and dnCXCL8, respectively. AP65, which incorporates the F17K/F21K substitutions, showed the strongest response, suggesting that the lysine substitutions may influence peptide–HLA binding affinity (34). Nevertheless, the difference in relative immunogenicity between AP65 and AP64 was not statistically significant. Interestingly, the relative donor-level differences between CXCL8 and dnCXCL8 proteins were not always mirrored at the peptide level. This discrepancy may reflect differences in antigen processing: intact proteins are subject to endosomal processing, generating a broad repertoire of epitopes, while synthetic peptides present only predefined sequences. Additionally, some protein regions not represented in the peptide panel may contribute to the higher overall donor reactivity observed for full-length proteins, despite their lower molar concentrations compared to peptides (43).

### 4.3 Mechanistic considerations

The modest increase in immunogenicity observed for AP65 could be mechanistically explained by the introduction of positively charged lysine residues at positions 17 and 21. These substitutions may alter peptide–MHC interactions, modify local protein solubility, or impact glycosaminoglycan (GAG) binding and multimerization, thereby indirectly affecting antigen presentation (44, 45). Further studies such as HLA-binding assays or structural modeling will be required to delineate these mechanisms.

### 4.4 Clinical relevance

From a translational perspective, the observation that dnCXCL8 does not display significantly increased immunogenicity compared to wild-type CXCL8 is encouraging. For biologics intended for chronic administration, even small increases in immunogenicity can be of concern. Our data indicate that dnCXCL8 maintains an immunogenic profile comparable to CXCL8, supporting its continued development as a therapeutic candidate. The broader finding that chemokines can induce measurable immune responses aligns with the concept that they naturally predispose to humoral autoreactivity. This underscores the importance of monitoring autoantibody and T-cell responses in clinical trials of chemokine-based therapeutics (46).

### 4.5 Limitations

Several limitations of this study should be mentioned. Recombinant proteins were expressed in *E. coli*, and even trace amounts of endotoxin can act as strong adjuvants through TLR4 signaling (47). All protein preparations were confirmed by Limulus Amebocyte Lysate (LAL) testing to contain <0.06 EU/mL, in line with internal specifications. Endotoxin contaminations can be avoided if protein expression is accomplished for example in yeast (Pichia Pastoris). In addition to endotoxins, high levels of TFA could influence immune cell activation. And although the manufacturer’s standard specifications were met, we cannot exclude that residual TFA contributed to some of the peptide responses. Finally, for peptide responses, a less stringent statistical threshold (p < 0.1) was applied to reduce false negatives, reflecting the exploratory nature of the assay. While suitable for early-stage screening, confirmatory studies under stricter criteria will be needed to validate these findings.

### 4.6 Future perspectives

Future work will extend this approach to additional engineered chemokines, which represent a promising novel class of anti-inflammatory biotherapeutics (5, 24). With respect to extending the indication range of our protein engineering approach, we are planning to extend our studies to CCL2 (monocyte/macrophage-mobilizing) and CXCL12 (T-cell/stem-cell mobilizing) dominant-negative mutants. Comparative studies with naturally occurring chemokine isoforms may provide further insights into determinants of immunogenicity. Moreover, mechanistic investigations into how specific amino acid substitutions influence epitope processing, HLA binding, and T-cell recognition will be critical to guide the rational design of safer and more effective chemokine-based therapeutics.

## Data Availability

The original contributions presented in the study are included in the article/**Supplementary Material**. Further inquiries can be directed to the corresponding author.

## Glossary

APC: Antigen-presenting cell
CEFT: CMV, EBV, Flu, Tetanus peptide mix
CXCL8: C-X-C Motif Chemokine Ligand 8
CXCR1/CXCR2: C-X-C Motif Chemokine Receptor ½
dnCXCL8: Dominant-negative CXCL8 mutant
DRB1: DRB3/4/5, DQ, DP, HLA class II loci
FACS: Fluorescence-activated cell sorting
FSC: Forward scatter
GAG: Glycosaminoglycan
GPCR: G protein–coupled receptor
HLA: Human Leukocyte Antigen
IFNγ: Interferon-gamma
IL-8: Interleukin 8
KLH: Keyhole Limpet Hemocyanin
LAL: Limulus Amebocyte Lysate
MFI: Mean Fluorescence Intensity
MHC: Major Histocompatibility Complex
PBS: Phosphate-buffered saline
PBMC: Peripheral Blood Mononuclear Cells
RP-HPLC: Reverse-phase high-performance liquid chromatography
RR: Relative Response
SI: Stimulation Index
SSC: Side scatter
TFA: Trifluoroacetic acid
TH (T-helper): CD4⁺ T-helper cell
TLR4: Toll-like receptor 4
TT: Tetanus Toxoid
wtCXCL8: Wild-type CXCL8
