# Supplementary material for: Immunogenicity of a CXCL8-based biopharmaceutical drug candidate in comparison to its wildtype form
Source: Front Immunol. 2025 Dec 16;16:1679409. doi: 10.3389/fimmu.2025.1679409 (PMC12747977; doi:10.3389/fimmu.2025.1679409)

Supplementary Material

# Supplementary Tables and Figures

**Supplementary data 1:** HLA allotypes that are considered for TH-epitope profiling. This table lists all HLA allotypes included in the Epibase® profiling. The frequencies of each allotype in the Caucasian population are given, alongside the corresponding serotypes. Because each individual carries two alleles per gene, total frequencies may exceed 100%. Data are expressed as percentages based on reference population data.


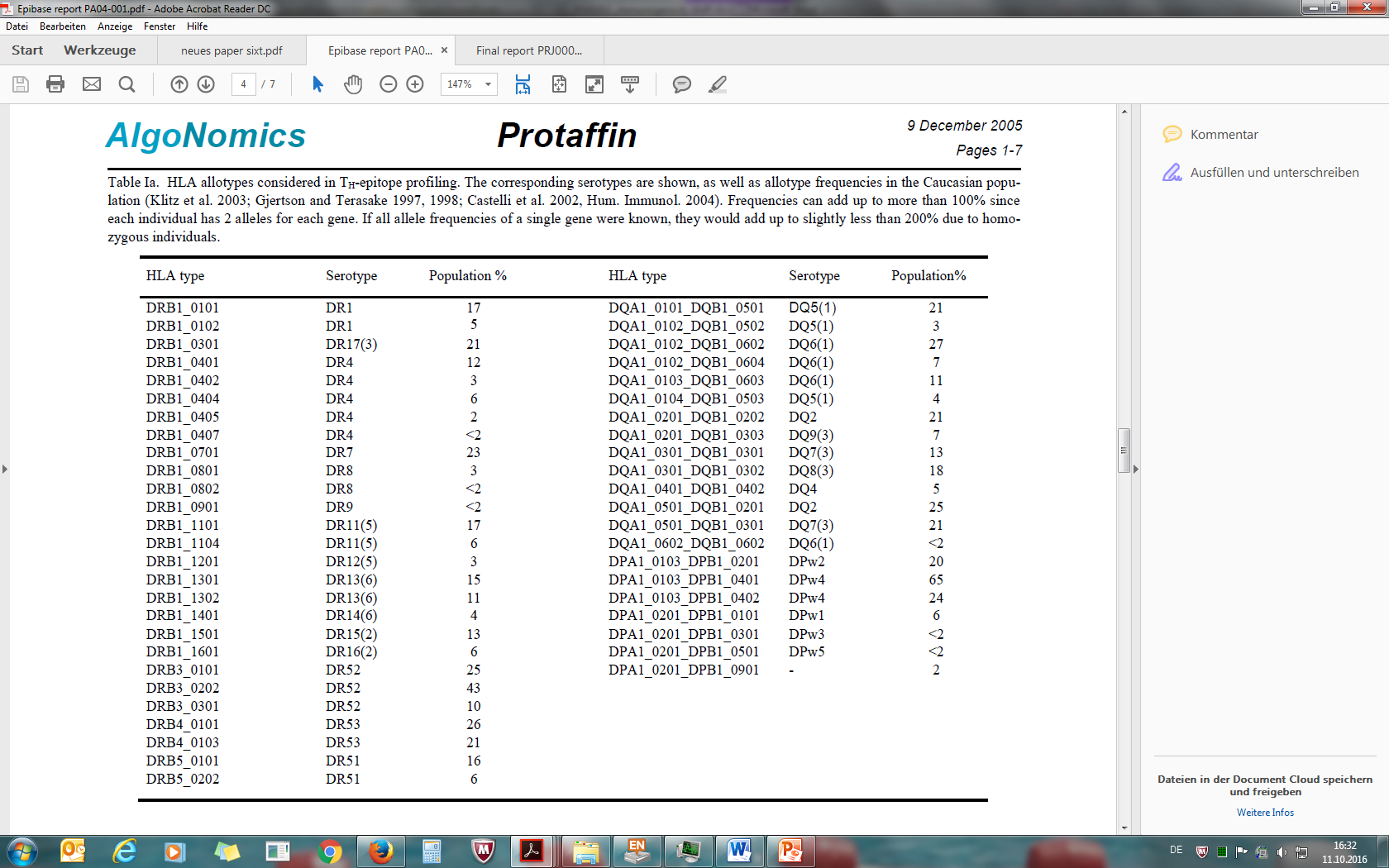


**Supplementary data 2:** List of variable parameters used in the automated classification tool and their description. The table provides an overview of the variable parameters included in the automated classification tool, together with their definitions. These parameters were used to distinguish activated versus non-activated wells in peptide-induced T-cell activation assays.


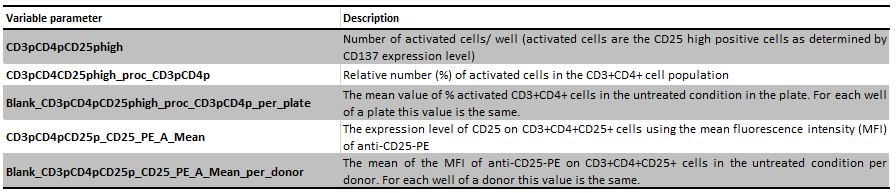


**Supplementary data 3:** Overview of strong TH-epitopes in CXCL8 and dnCXCL8. Epitope mapping results are displayed separately for CXCL8 (3.1) and dnCXCL8 (3.2). Predicted strong CD4⁺ T-helper epitopes were identified using the Epibase® algorithm across the full amino acid sequences. The table indicates peptide sequences, start/end positions, implicated HLA allotypes, and prediction scores. Data are displayed as predicted binding strengths.

3.1 Overview of strong TH-epitopes in CXCL8:

3.2 Overview of strong TH-epitopes in dnCXCL8:

**Supplementary data 4:** Table of SI values for KLH and TT with corresponding p-values and 95% confidence intervals (CI) for all donors. The table shows stimulation index (SI) values, 95% confidence intervals (CI), and p-values for each donor tested with KLH and TT. Each donor was tested in 10 replicates per antigen. Data are displayed as mean SI ± SEM.


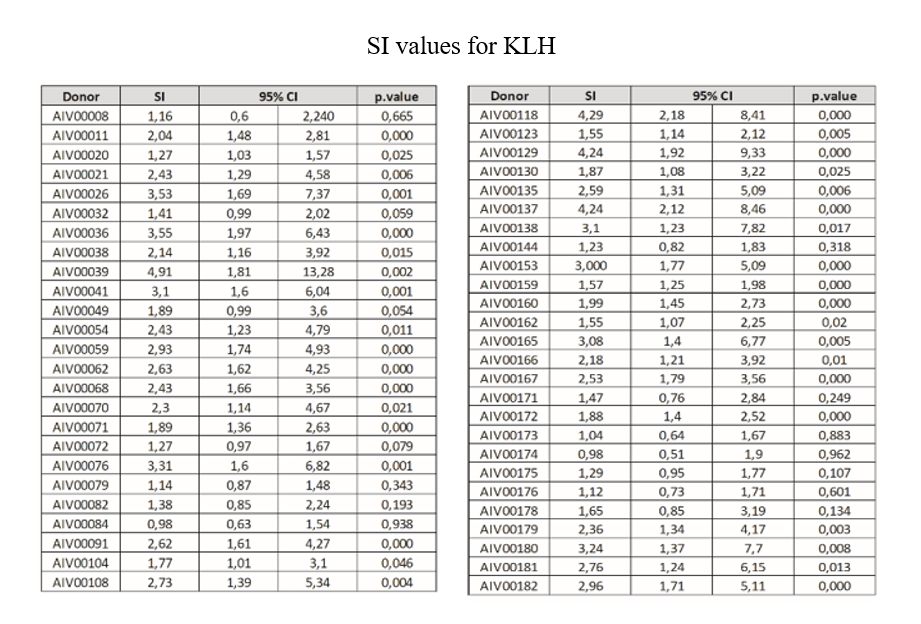


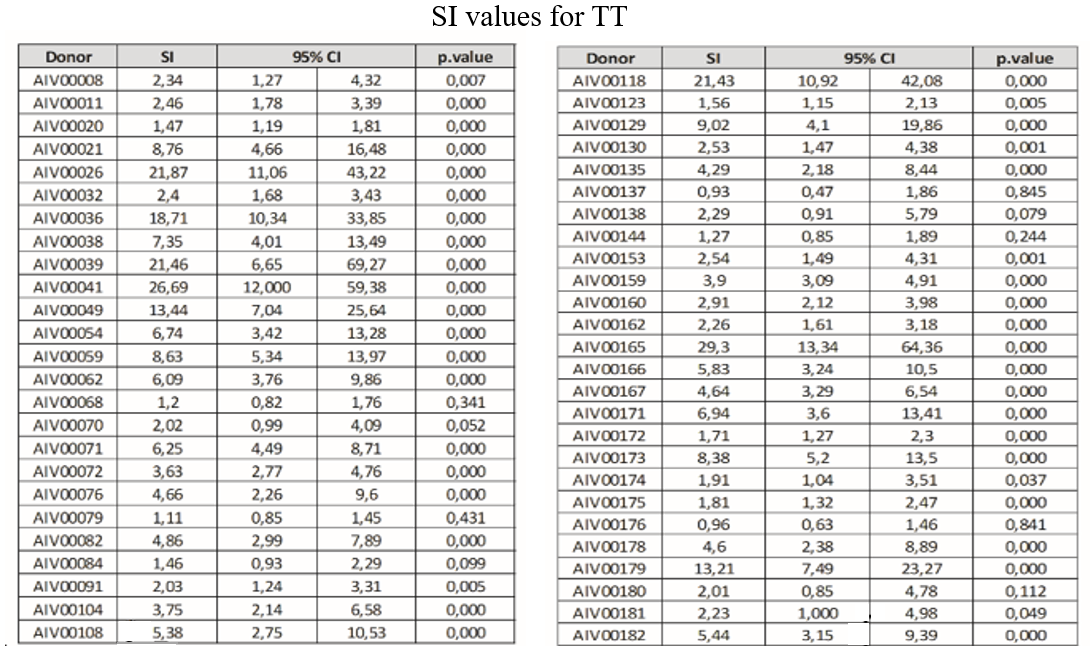


**Supplementary data 5:** Table of SI values for CXCL8 with corresponding p-values and 95% confidence intervals (CI) for all donors. Table presents stimulation index (SI), 95% confidence intervals (CI), and p-values for each donor in response to CXCL8. Each donor was tested in 10 replicate wells. Data are expressed as mean SI ± SEM.


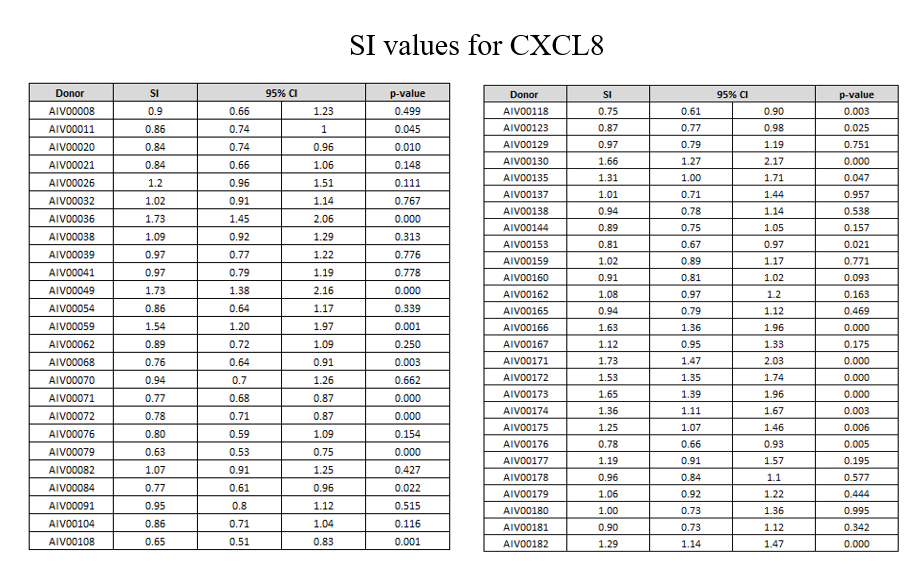


**Supplementary data 6**: Donor-level immunogenicity responses to KLH. Log-transformed stimulation index (SI) values for Keyhole Limpet Hemocyanin (KLH) compared to blank wells are shown for individual donors (n = 51). Each point represents the mean SI for one donor, with error bars indicating the 95% confidence interval. (*) denote donors with a significant immunogenic response (SI ≥ 1.5, p ≤ 0.05). This figure illustrates the distribution of KLH-induced CD4⁺ T-cell activation across the donor population.


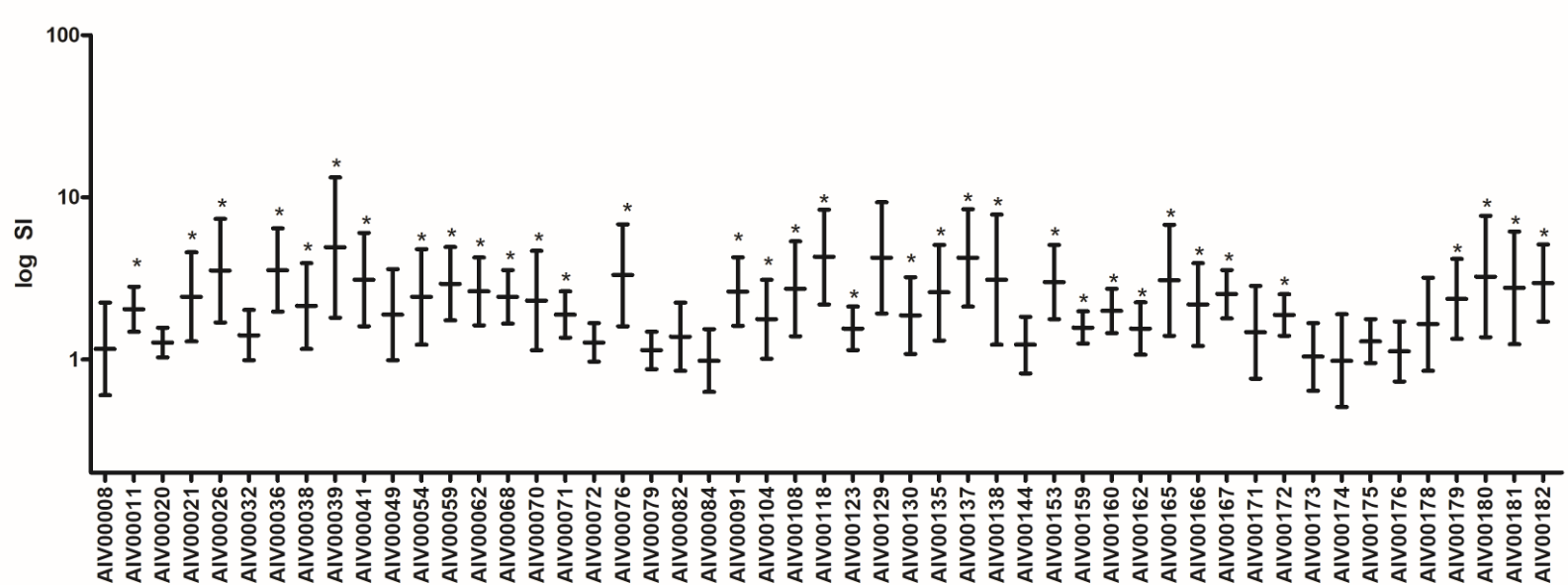


**Supplementary data 7:** Donor-level immunogenicity responses to Tetanus toxoid (TT). Log-transformed stimulation index (SI) values for TT compared to blank wells are displayed for each donor (n = 51). Each data point shows the mean SI, with error bars indicating the 95% confidence interval. (*) mark donors with a significant immunogenic response (SI ≥ 1.5, p ≤ 0.05). TT served as a positive control antigen and demonstrated robust immunogenicity across the majority of donors..


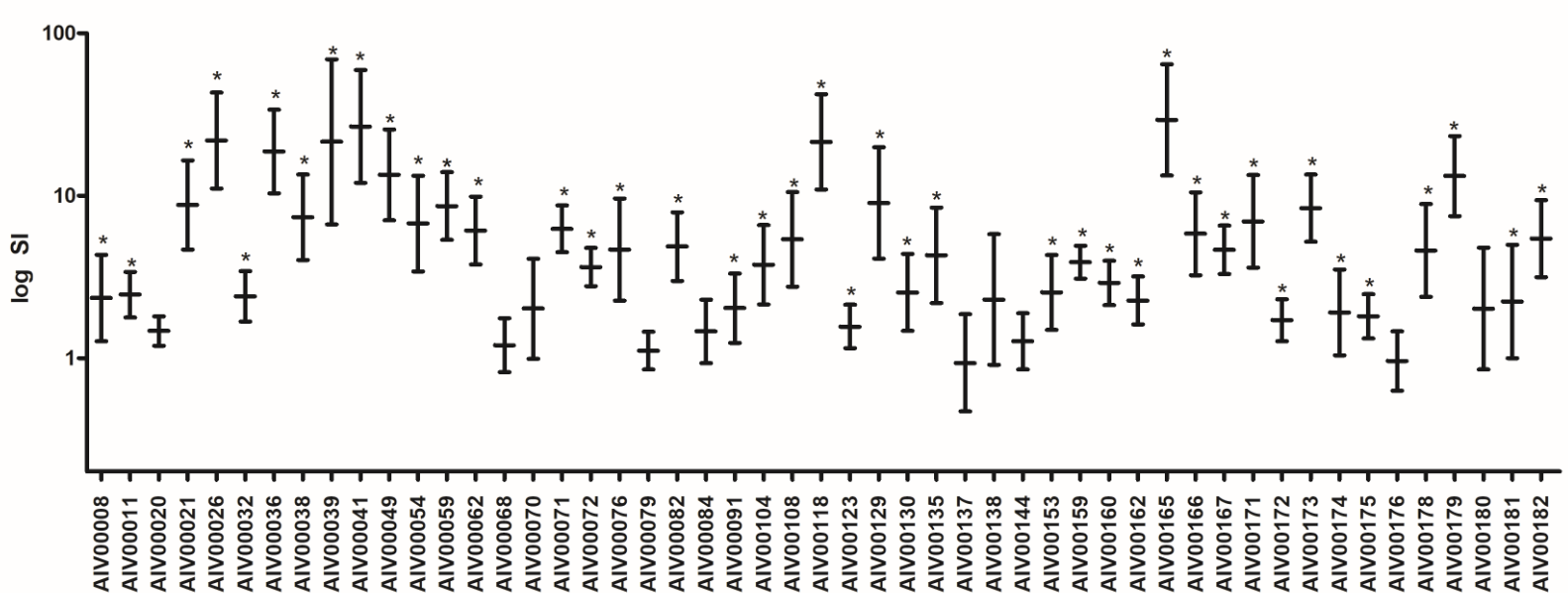


**Supplementary data 8**: Donor-level immunogenicity responses to CXCL8. Log-transformed stimulation index (SI) values for wild-type CXCL8 compared to blank wells are shown for each donor (n = 51). Data are presented as mean SI values with 95% confidence intervals. Donors meeting the significance threshold (SI ≥ 1.5, p ≤ 0.05) are indicated with (*). This figure shows that CXCL8, despite being a self-protein, elicited detectable immunogenic responses in a subset of donors.


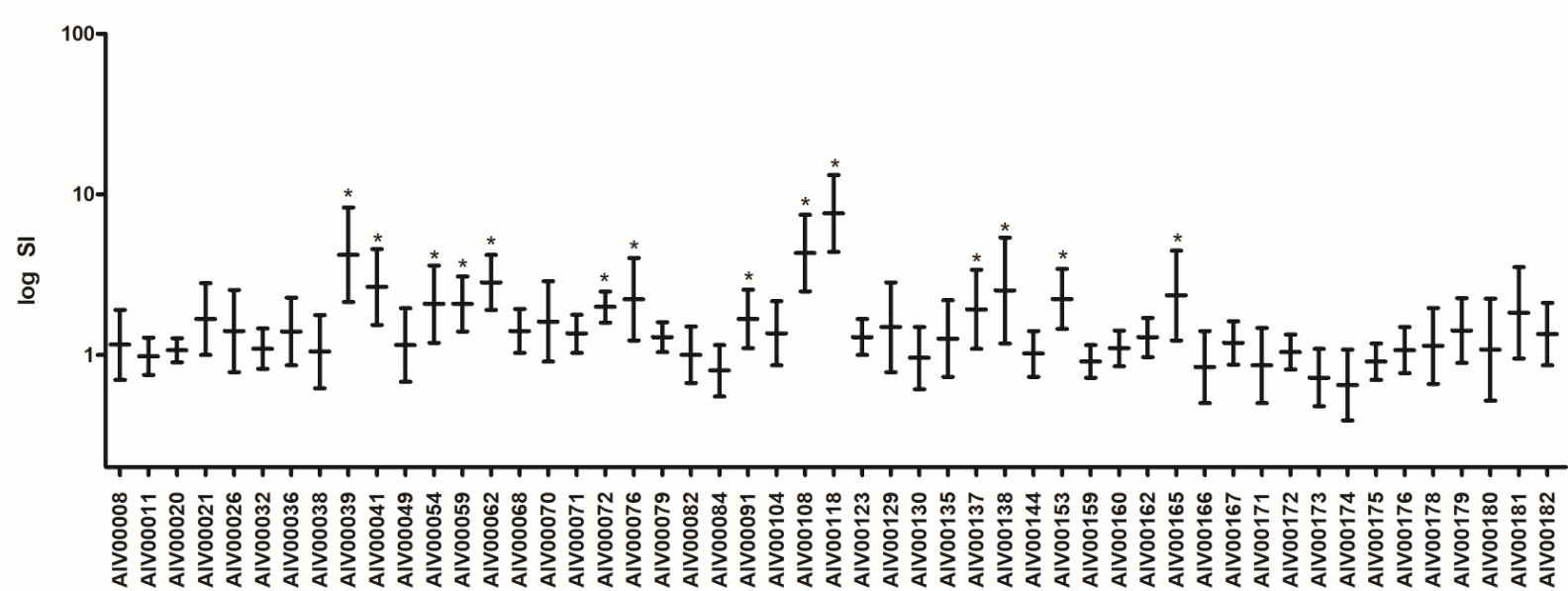


**Supplementary data 9:** Donor-level immunogenicity responses to dnCXCL8. Log-transformed stimulation index (SI) values for the dominant-negative CXCL8 mutant (dnCXCL8) compared to blank wells are presented for individual donors (n = 51). Each value represents the mean SI, with error bars indicating the 95% confidence interval. Significant donor responses (SI ≥ 1.5, p ≤ 0.05) are denoted by (*). This figure highlights that dnCXCL8 elicited immunogenicity in a slightly higher number of donors compared to CXCL8, though the difference was not statistically significant at the population level.


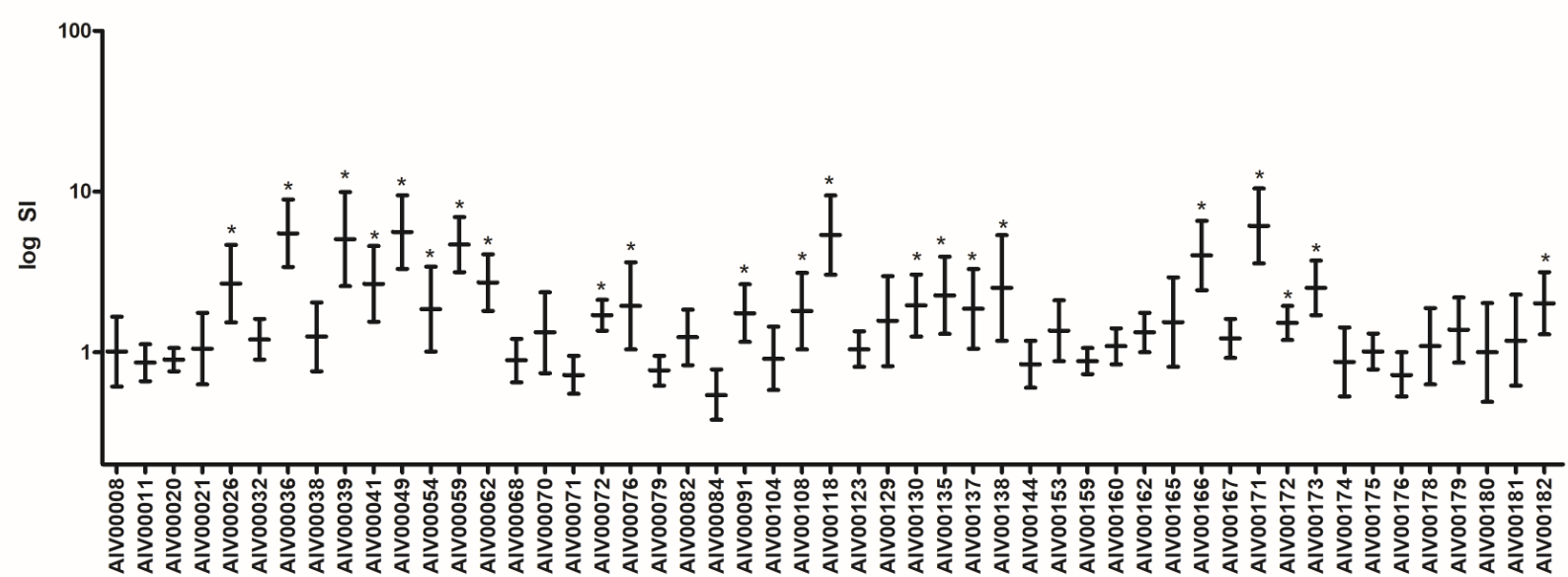

Supplement: Supplementary file 1 [file DataSheet1.docx]
